# Prefrontal control of attention to threat

**DOI:** 10.3389/fnhum.2013.00024

**Published:** 2013-02-05

**Authors:** Polly V. Peers, Jon S. Simons, Andrew D. Lawrence

**Affiliations:** ^1^MRC Cognition and Brain Sciences UnitCambridge, UK; ^2^Department of Psychology, University of CambridgeCambridge, UK; ^3^School of Psychology, Cardiff UniversityCardiff, UK

**Keywords:** anxiety, attentional blink, biased competition, cognitive control, emotion, facial expression, fMRI, prefrontal cortex

## Abstract

Attentional control refers to the regulatory processes that ensure that our actions are in accordance with our goals. Dual-system accounts view temperament as consisting of both individual variation in emotionality (e.g., trait anxiety) and variation in regulatory attentional mechanisms that act to modulate emotionality. Increasing evidence links trait variation in attentional control to clinical mood and anxiety disorder symptoms, independent of trait emotionality. Attentional biases to threat have been robustly linked to mood and anxiety disorders. However, the role of variation in attentional control in influencing such biases, and the neural underpinnings of trait variation in attentional control, are unknown. Here, we show that individual differences in trait attentional control, even when accounting for trait and state anxiety, are related to the magnitude of an attentional blink (AB) following threat-related targets. Moreover, we demonstrate that activity in dorso-lateral prefrontal cortex (DLPFC), is observed specifically in relation to control of attention over threatening stimuli, in line with neural theories of attentional control, such as guided activation theory. These results have key implications for neurocognitive theories of attentional bias and emotional resilience.

## Introduction

Facial expressions provide critical information about potential threat. For example, angry expressions convey aggressive intent or disapproval, and fearful expressions convey the presence of environmental danger (Whalen, 1998). Accordingly, it is thought that threat-related faces receive a prioritized access to limited cognitive resources (Vuilleumier, 2005).

A normative function of such attentional prioritization is to help the organism respond effectively to significant danger (Lazarus, 1991). However, exaggerated biases in processing innocuous threat-related information are implicated in the etiology and maintenance of mood and anxiety disorders. Specifically, it has been suggested that the attentional system of clinically anxious individuals may be distinctively sensitive to and biased in favor of threat-related stimuli in the environment (Mathews and Mackintosh, 1998; Mogg and Bradley, 1998). While attentional bias to threat (i.e., differential attentional allocation toward threatening vs. neutral stimuli) is a robust finding in anxious populations (Bar-Haim et al., 2007 for meta-analysis), the mechanisms underpinning human variation in such bias remain unclear.

Perhaps unsurprisingly, given the well-established link between the emotional traits of neuroticism and behavioral inhibition and mood disorders (Kotov et al., 2010 for meta-analysis), much of the experimental work examining individual variation in attentional bias for threat has focused on trait negative emotionality. This work has shown increased attentional bias to threat in high anxious, non-clinical individuals (Bar-Haim et al., 2007 for meta-analysis). Likewise, neuroimaging studies of individual differences in facial threat processing have focused on individual differences in negative emotionality (e.g., trait anxiety, harm avoidance). Such studies have generally observed enhanced activity in the amygdala [a structure known to be critical for fearful responding and fear learning, (Johnson et al., 2009)] to unattended threat-related faces and scenes with increasing anxiety (Bishop et al., 2004; Ewbank et al., 2009) or harm avoidance (Most et al., 2006); and have led to the development of neuro-cognitive models positing a key role for the amygdala in mediating attentional bias (Vuilleumier, 2005).

According to “dual system” accounts, temperament is not only composed of individual variation in emotional reactivity (e.g., trait anxiety) but also comprises dispositional differences in self regulatory control mechanisms that act to modulate emotional reactivity (Posner and Rothbart, 2009). Trait attentional control reflects stable individual differences in the efficiency of executive attention. Key aspects of trait attentional control include the ability to flexibly control attentional allocation and to effortfully maintain or disengage attention (Posner and Rothbart, 2009; Bridgett et al., 2012).

There is increasing evidence that variation in attentional control prospectively predicts the development and maintenance of mood and anxiety disorders, both independently of and in interaction with negative emotionality (Oldehinkel et al., 2007; Verstraeten et al., 2009; Sportel et al., 2011; Van Oort et al., 2011; Yap et al., 2011). Further, twin studies show shared genetic influences on both trait attentional control and mood and anxiety symptoms (Lemery-Chalfant et al., 2008), suggesting links at an etiological level. Despite the clear protective effect of attentional control on mood and anxiety symptomatology, previous research on threat-related processing has largely neglected the role of individual variation in attentional control in attenuating attentional bias to threat (but see Derryberry and Reed, 2002; Lonigan and Vasey, 2009) and in influencing prefrontal attentional control mechanisms (but see Gyurak et al., 2012). Recently, Cisler and Koster (2010) suggested that poor attentional control may be a potential mechanism mediating certain elements of attentional bias for threat, in particular, difficulties in “disengaging” attention from threat. Attentional dwell time paradigms, which allow measurement of the (dis)engagement of attentional resources from an initial target, by examining its impact on identification of a subsequent target, represent an ideal paradigm to test this hypothesis (Ward et al., 1997).

An extensively studied effect in the literature on rapid serial visual presentation (RSVP) is the attentional blink (AB) (Raymond et al., 1992). In studies of the AB a deficit in the identification of a second target, (T2), is typically observed, if that target appears in a rapid stream of distractors within ~500 ms of an initial target (T1). The AB is thought to arise from attentional demands of T1 for selection, working memory encoding, episodic registration, and response selection, which prevents this high-level central resource from being applied to the second target when the time between the presentation of T1 and T1 (T1–T2 lag) is short (Dux and Marois, 2009). T1 processing also transiently impairs the redeployment of these attentional resources to subsequent targets (Dux and Marois, 2009). Recent studies show that a threat-related or negative T1 target (e.g., an angry face) relative to a neutral T1 can lead to an enhanced AB (i.e., greater difficulty in reporting the T2 identity) for a subsequent neutral T2 target (Mathewson et al., 2008; Koster et al., 2009; de Jong et al., 2010). If variation in regulatory temperament is important in controlling the bias toward threatening stimuli, then individual variation in attentional control should predict the impact of threat-related T1 stimuli on subsequent neutral T2 identification (i.e., the magnitude of the threat-related AB). Indeed, recently, we demonstrated behaviorally that individuals with poor attentional control showed impaired target processing in an RSVP task following presentation of an irrelevant emotional distractor, if the target appeared within 200 ms of the distractor (Peers and Lawrence, 2009). However, the neural basis of this effect remains unclear.

To bypass the sluggish temporal resolution of fMRI, here we move away from the standard AB paradigm and instead use the closely related 2-target paradigm, known to tap a common attentional limitation (Ward et al., 1997; Dux and Marois, 2009). In addition, T1 and T2 target stimuli were selected from different visual categories (faces and scenes, respectively) that activate anatomically distinct regions—fusiform face area (FFA) (Kanwisher et al., 1997), and parahippocampal place area (PPA) (Epstein and Kanwisher, 1998). This allowed us to examine, for the first time, the brain regions mediating the influence of variation in trait attentional control on the magnitude of the threat-related AB.

We predicted that weaker attentional control would be associated with an enhanced AB following a threat-related relative to a neutral T1 (Peers and Lawrence, 2009). Further, we predicted that regions of prefrontal cortex implicated in top-down attentional control (Miller and Cohen, 2001; Duncan, 2010) would mediate the influence of variation in trait attentional control on the magnitude of the threat-related AB. Specifically, we predicted that individuals with better attentional control would show greater prefrontal cortex activity on trials in which threatening information was successfully inhibited. Given the proposed role of the amygdala in prioritizing threat-related material (Vuilleumier, 2005), AB for threat may also be related to heightened amygdala activity. It is possible that any amygdala activity associated with the threat AB could also correlate with anxious temperament. This potential effect in the amygdala may occur either in addition to any attentional control effects or in the absence of such effects of control (Mathews et al., 2004; Bishop et al., 2007).

## Materials and methods

### Participants

Nineteen healthy volunteers (9 female, all right-handed, aged 19–40, mean age 27 years) with normal, or corrected to normal, vision participated. No participant had a history of neurological disease or head injury or was currently on medication affecting the CNS. One additional participant was excluded due to scanner malfunction. The study was approved by Suffolk Local Research Ethics Committee. All volunteers provided written informed consent and received a small honorarium.

Participants were selected from an initial sample of 55 volunteers who had completed a number of mood and personality questionnaires. These included the attentional control scale (ACS) (Derryberry and Reed, 2002), which contains 20 items such as “When I am trying to focus my attention, I am easily distracted” (reverse scored), rated on a four point Likert scale from 1 (almost never) to 4 (always); and the trait anxiety subscale of the Hospital Anxiety and Depression Scale (HADS) (Zigmond and Snaith, 1983) (seven items, score range 0–21). Two recent studies (Sulik et al., 2009; Bridgett et al., 2012) have demonstrated that both self-report measures of attentional control and performance on cognitive control tasks like the Stroop task are indicators of a single latent attentional control construct. The HADS anxiety scale has excellent reliability and correlates highly with other measures of trait anxiety such as the Spielberger (1983) Trait Anxiety Inventory (Bjelland et al., 2002) and the Carver and White (1994) Behavioral Inhibition Scale (BIS) (Brunborg et al., 2010). Selection from this sample was carried out on the basis of scores on these scales to ensure a range of attentional control and anxiety scores in the fMRI sample. ACS scores ranged from 45 to 70 (mean 57.1, SD 6.2) whilst HADS anxiety scores ranged from 2 to 18 (mean 5.5, SD 2.5) and were comparable to published norms of healthy populations (Crawford et al., 2001).

Immediately prior to scanning, participants completed a measure of state anxiety—the State form of the Spielberger State-Trait Anxiety Inventory (STAI) (range, 21–42, mean 29.92, SD 6.88) (Spielberger, 1983).

### Task

**Figure 1:**
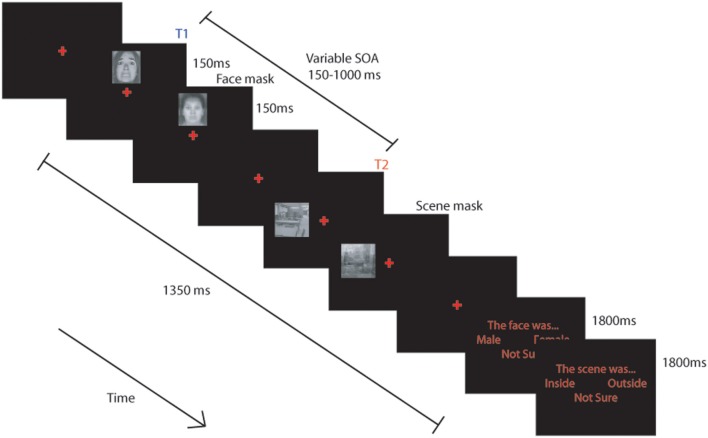
**Schematic representation of the task used.** BOLD signal was modeled from the onset of each trial and included the response phase. (see text for details).

We modified the 2-target attentional dwell task, (based on Ward et al., 1997), to examine the influence of individual differences in attentional control and state/trait anxiety on the allocation of attentional resources to threat-relevant (fearful) and neutral initial targets (T1), based on their impact on processing of a subsequent neutral T2 target (scene) following closely in time. Trials comprised a single masked fearful or neutral face followed by a single masked neutral scene (T2) presented in unpredictable locations (Figure 1).

### Stimuli

Two classes of stimuli were used, faces and scenes, which have been shown to selectively activate distinct brain areas—FFA (Kanwisher et al., 1997) and PPA (Epstein and Kanwisher, 1998), respectively. The use of these classes of stimuli allowed us to localize the neural responses of the T1 and T2 stimuli and to assess the BOLD response to the T2 stimulus uncontaminated by T1 activity. The face stimuli were taken from two standardized databases: the Pictures of Facial Affect (POFA) and Caucasian images from the Japanese and Caucasian Facial Expressions of Emotion (JACFEE) (www.paulekman.com). They comprised eight females and seven males displaying both neutral and fearful expressions. Scene stimuli were selected from a large database of pictures of visual scenes which have previously been shown to evoke activation in regions of “parahippocampal place area” (PPA) (Epstein and Kanwisher, 1998). The stimulus set comprised 15 black and white images of “inside” locations and 15 images of “outside” locations. The “outside” locations were a mixture of natural landscapes (seven scenes) and urban locations (eight scenes). Face and scene masking stimuli were developed by superimposing examples of the faces or scenes on top of one another to produce a stimulus with low level contours, resembling the stimulus category, but which did not look like any of the individual items specifically. Previous work, with face stimuli at least, (Peers et al., 2005) has shown that this type of mask has similar psychometric properties to that of a pattern mask used with letters.

### Procedure

The experiment was run on a Dell desk-top computer. The participants viewed the stimuli via a Christie video projector seen through a mirror positioned 90 mm from their eyes. Participants' responses were collected using a 4-button serial response box. Experiments were programmed using E-prime (Psychology Software Tools, Inc.).

### Block structure

The scanning session comprised 4 blocks of 128 trials. Each block was separated into three task conditions: either a single task “attend face only” condition, a single task “attend scene only” condition or a dual task, “attend both face and scene” condition. The single task conditions were included to ensure participants could selectively attend and that we could reliably detect FFA and PPA activity in this paradigm but are not discussed further. Each block of trials started with a sub-block (32 trials) of one of the single task conditions followed by two sub-blocks (64 trials) of the dual task condition, followed by a sub-block of the other single task condition. All participants completed the “attend face only” condition first in blocks 1 and 4 and the “attend scene only” condition first in blocks 2 and 3. Instructions displayed at the beginning of each task informed participants of the task to be completed. These appeared on the screen for 5 s with arrows pointing to the possible locations of targets and instructed the participant to “attend face,” “attend scene,” or “attend face and scene” (Figure 1).

### Trial structure

Each trial followed essentially the same pattern, with only the initial instruction and the response requirements manipulated across conditions. In the single task conditions participants were requested to attend only to either the face or the scene. When they were to attend to the face they were requested to indicate whether it was male or female and when they were to attend to the scene they were to indicate whether it was inside or outside. Trials commenced with presentation of a red central fixation cross (~0.7° × 0.7°) on a black screen for a variable duration between 150–300 ms. A target face stimulus (~2.5° × 3.2°) was then presented randomly ~2.3° above or below the cross for 150 ms before being replaced by the face mask for 150 ms. After an stimulus onset asynchrony (SOA) of 150, 300, 450, or 1000 ms following presentation of the face, a scene (~3.2° × 3.2°) was presented randomly ~3° to the left or right of the central cross for 150 ms before being replaced by a scene mask for an equivalent duration. The experiment was fully counterbalanced for face gender (male and female) and expression (fearful or neutral), scene location (inside or outside) and SOA.

### Task demands

In the “attend face only” and “attend face and scene” conditions, a response screen was presented 1350 ms after the onset of the face stimulus, which instructed participants to press the leftmost button to indicate a male face was present, the rightmost button to indicate a female face, and either of the central buttons if they were “not sure.” The response screen was displayed for 1800 ms. The response screen for the scenes (in the “attend scene only” and “attend face and scene” conditions), presented 3150 ms after the onset of the face, instructed participants to press the leftmost button to indicate that the scene was “inside,” the rightmost button for “outside” scenes, and either of the central buttons if they were “not sure.” Responses were collected for both decisions during the 1800 ms presentation of the response screen. A 500 ms rest period was provided between trials.

Participants were instructed to only attend to the stimuli indicated by the task instruction, and to try to ignore other stimuli. They were informed that on each trial the face would appear first either above or below the cross and that the scene would then appear either to the left or right. They were told that in dual task trials they would always be requested to make the decision about the face before the decision about the scene and were requested to respond to items only when the response cue was present.

All participants attempted a short version of the task outside the scanner on a separate visit and were then given eight trials of practice on each of the tasks on the day of scanning outside the scanner.

### Localizer

An independent localizer scan was performed in the same participants to define FFA and PPA at the end of the scanning session. Participants were required to perform a 1-back matching task, in which four 16 s blocks of each stimulus type (scenes, neutral faces, fearful faces, and objects) were presented in a pseudorandom order. Each block consisted of 20 stimuli (18 different images and two repeats) presented centrally on the screen for 300 ms with a 500 ms gap between stimuli. Images were selected from those used in the dwell time task, with additional faces drawn from the NimStim Face Stimulus Set (Tottenham et al., 2009). Object stimuli were selected from a set of objects previously used in localizer tasks (Epstein and Kanwisher, 1998). Participants were instructed to view each of the stimuli and to press any button when they saw an immediate repetition of an image.

### Image acquisition

MRI scanning was performed on a Siemens Tim Trio 3-Tesla MR scanner. Whole brain data were acquired with T2^*^-weighted echo-planar imaging (EPI) sensitive to blood oxygenation level-dependent (BOLD) contrast. Each image volume consisted of 32 sequentially acquired axial oblique 3 mm thick slices (interslice gap = 25%; FOV = 192 mm × 192 mm; matrix size = 64 × 64; flip angle = 78°; voxel bandwidth 2232 Hz/Px; TE 30 ms; TR 2000 ms). Four functional runs of the dwell time task, each of 380 volume acquisitions, were acquired together with one localizer run of 154 volumes. The first six volumes of each run were discarded to allow for T1 equilibration. T1 weighted structural images were acquired at a resolution of 1 mm^3^.

### Image analysis

Data were pre-processed and analyzed using SPM5 (Wellcome Trust Centre for Neuroimaging, London, UK). Functional images were first corrected for motion by realigning all images with respect to the first, and for differences in slice timing by re-sampling all slices in time to match the middle slice. Each participant's structural image was co-registered to the mean of the realigned functional images and then segmented to separate out gray matter, which was normalized to the gray matter in a template image in MNI stereotactic space. The realigned EPI images were then spatially normalized using the structural normalization parameters, re-sampled into 3 mm cubic voxels and spatially smoothed with an 8 mm FWHM isotropic Gaussian kernel. A high-pass filter of 1/128 Hz was used to remove low-frequency noise, and an AR(1) model corrected for temporal autocorrelations.

Random effects statistical analysis was undertaken in two stages. In the first stage, event types for each functional run were modeled by convolving onset times of trials with a canonical hemodynamic response function. Thus, the BOLD signal for each trial captured both presentation and behavioral response. For the localizer task, a block-design was used. Parameters for each regressor were estimated using a subject-specific model, with movement parameters in the three directions of motion and 3° of rotation included as confounds, and covariates representing the mean session effects. Linear contrasts were used to obtain subject-specific estimates for each of the effects of interest. These estimates were entered into the second stage of analysis treating subjects as a random effect, using one-sample *t*-tests across subjects. Additionally, regression analyses were carried out using participants' attentional control scores as a covariate.

**Figure 2:**
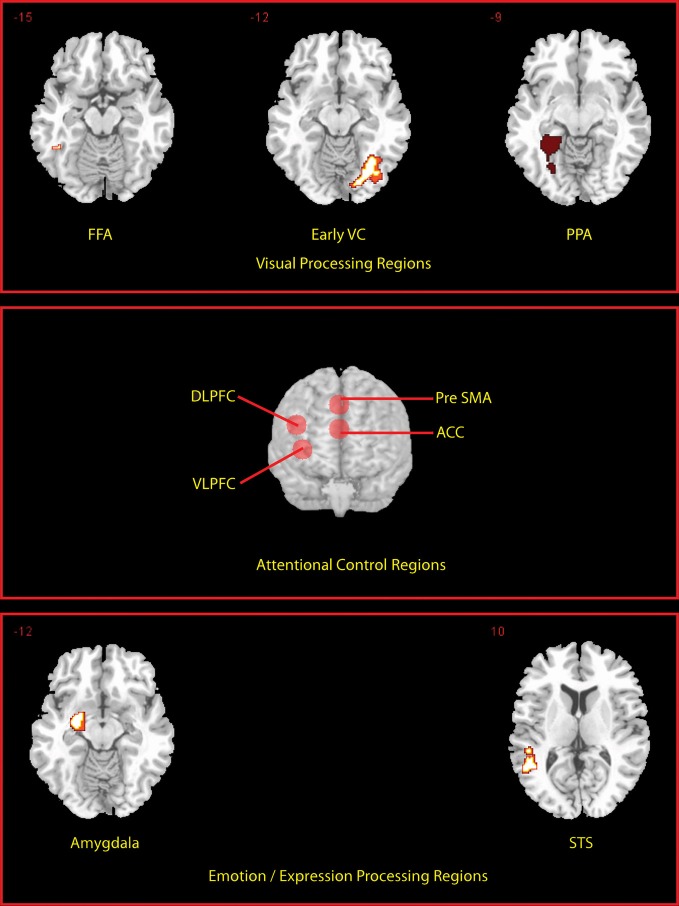
**Locations of a priori defined ROIs.** Transverse slices for the peak voxel of each of the fROI's are shown. Prefrontal ROIs are rendered on to the anterior surface of a whole brain.

We defined a priori regions of interest (ROIs) (see Figure 2) independently of the data under examination, based on a separate localizer scan or on coordinates reported in previous studies. Functional localizer ROIs (fROIs) (Kawabata Duncan and Devlin, 2011) for the fusiform face area (FFA; (176 mm^3^ in extent) parahippocampal place area (PPA; left PPA 2304 mm^3^, right PPA 3165 mm^3^), superior temporal sulcus (STS; 2832 mm^3^), and amygdala (2104 mm^3^) were defined as the group-level peak clusters nearest the previously published co-ordinates for these regions in the independent localizer contrasts (faces > scenes and objects) (for FFA and amygdala), (fear faces > neutral faces) (for the STS) and (scenes > faces and objects) (for the PPA)^1^. These were created using the MarsBar toolbox (Brett et al., 2002). In addition an early visual cortex (earlyVC; 729 mm^3^) f-ROI was created in the same way using a comparison of all visual events in the localizer against rest. Prefrontal ROIs sampled using 10 mm radius spheres centered on coordinates based on a meta-analysis of multiple-demand regions (Duncan, 2010) were produced for dorso-lateral prefrontal cortex; DLPFC center (±42, 24, 25), ventrolateral prefrontal cortex; VLPFC (±36, 18, 1), anterior cingulate; ACC, (0, 31, 21), and pre-supplementary motor area; pre-SMA (0, 20, 45). Activations are reported if they exceeded the family-wise error threshold of *p* < 0.05 small volume correction (SVC) for ROIs. Activations occurring outside the ROI were reported if they exceeded the family-wise error threshold of *p* < 0.05 whole-brain corrected and were larger than 10 voxels in extent.

## Results

### Behavioral results

#### T1 performance

Proportion of correct reports of T1 target gender did not differ as a function of expression *t* = −0.69, df = 18, *p* = 0.50, with values of 0.73, and 0.74 for neutral and fearful faces, respectively. To ensure that T1 had been attended to, subsequent behavioral and fMRI analyses were restricted to trials in which T1 had been correctly reported (T2|T1).

#### Influence of emotion and attentional control on T2 performance

**Figure 3:**
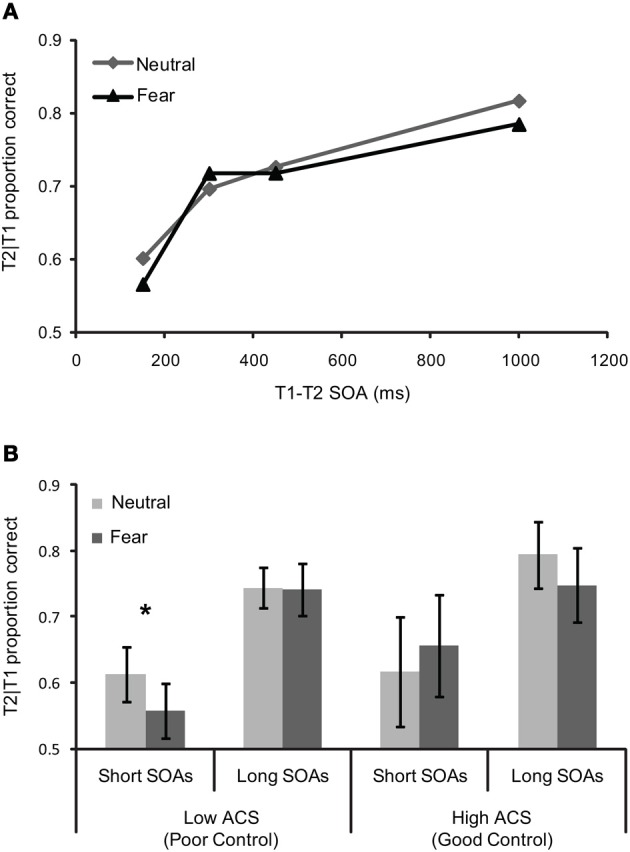
**Behavioral performance for (A) whole sample (*n* = 19)—T2 scenes task in the dual task condition as a function of the SOA between T1 (the face) and T2 (the scene), and (B) mean T1/T2 performance (±S.E.) for short and long SOAs as a function of T1 expression in high (*n* = 7) and low ACS (*n* = 7) groups.** The significant effects are marked with an asterisk.

The temporal dynamics of attention were assessed using a repeated-measures ANCOVA with T1 expression (neutral vs. fearful) and T1–T2 SOA (150, 300, 450, and 1000 ms) as a function of ACS score (Figure 3A). This revealed a near significant effect of SOA *F*_(3, 51)_ = 2.45.18, pη^2^ = 0.13, *p* = 0.07, as well as a significant T1 expression by SOA, *F*_(3, 51)_ = 2.83, pη^2^ = 0.14, *p* = 0.048 interaction and crucially a three-way expression by SOA by ACS interaction, *F*_(3, 51)_ = 3.12, pη^2^ = 0.16, *p* = 0.034, indicating a robust AB, an enhanced blink for threat relative to neutral faces, and a change in the profile of the blink associated to neutral and fearful faces with ACS score.

“High” and “low” ACS groups, based on median splits (with five participants falling on the median removed from the sample), were used to explore the three-way interaction. Trials were separated in to “short” (150, 300 ms) and “long” (450, 1000 ms) SOAs based on our previous findings that attentional control effects in RSVP were confined to SOA's less than 400 ms, (Peers and Lawrence, 2009). More broadly, interference effects in the dwell time paradigm are limited to T1–T2 SOAs of 400 ms or less, see Ward et al. (1997), Dux and Marois (2009). Paired samples *t*-tests comparing T2 accuracy for fearful vs. neutral face trials at “short” and “long” SOAs were carried out separately. These revealed a significant effect in the low ACS group at short SOAs *t* = −3.09, df = 6, *p* < 0.05, but no equivalent effect in the high ACS group *t* = 0.81 df = 6, *p* = 0.45, and no effect in either group at long SOAs (low ACS *t* = −0.08 df = 6, *p* = 0.94, high ACS *t* = −1.75, df = 6, *p* = 0.13), confirming a deeper AB for negative than for neutral T1 faces in the low ACS group only (Figure 3B). Corroborating the median split analysis, in the entire sample a significant correlation was observed between ACS score and threat-related relative to neutral AB magnitude at short lags *r* = 0.61, df = 17, *p* < 0.01.

#### Influence of emotion and trait and state anxiety on T2 performance

No significant correlations were observed between ACS and either state or trait measures of anxiety (STAIs, *r* = −0.32, *p* = 0.18; HAD-A, *r* = −0.04, *p* = 0.87) allowing us to examine effects of anxiety separately from those of ACS. Repeated-measures ANCOVAs carried out using STAI or HAD-A as covariates revealed significant effects of SOA but no other main effects or interactions, suggesting these anxiety measures did not influence performance. Crucially, the correlation between ACS and threat-related relative to neutral AB at short SOAs remained even when controlling for either STAIs (*r* = 0.61, df = 16, *p* < 0.01) or HAD-A (*r* = 0.65, df = 16, *p* = 0.01). Furthermore, HAD-A did not moderate the relationship between ACS and the threat relative to neutral AB at short SOAs (*t* = 0.21, df = 15, *p* = 0.84).

### fMRI data

Behaviorally we observed a modulation of the AB to threat related faces by trait attentional control. The key aim of our study was therefore to examine the neural correlates underpinning T2 interference from T1 threat and the effect of trait attentional control in modulating this, and as such we focus our results on the relevant contrasts specified below. Behavioral performance on the task provides a means for us to compare neural activity associated with successful disengagement from the T1 stimulus (i.e., “unblinked” trials or hits, when both T1 and T2 are correctly reported) with unsuccessful disengagement from T1 (“blinked” trials or misses). Data for short and long SOAs were collapsed as there were few blinked responses at long lags and thus any analyses would be underpowered.

### ROI based analyses

Below we report analyses carried out in each of our pre-defined ROIs, including the prefrontal (DLPFC, VLPFC, ACC, pre-SMA) regions, the category-selective FFA and PPA, and the amygdala and STS. Only significant results are reported.

#### Main effect of (un)successful dwell performance

No significant activity differences were seen in any of our ROIs when T2 hits vs. misses were compared for either the neutral-face or fear-face T1 conditions or no effects of attentional control were observed. The reverse contrast (misses vs. hits i.e., “attentional blinks”) showed no differences in the neutral-face condition. However, increased activity in the pre-SMA (*x*, *y*, *z* = 9, 21, 42, *Z* = 3.69, Psvc = 0.009) was seen for fear-face misses vs. hits. A previous study (Yeung et al., 2006) of task switching found that activity in pre-SMA increased as a function of interference between tasks. Similarly here the increased pre-SMA activity may reflect increased demands on conflict or error monitoring processes when the fearful face disrupts T2 performance.

#### Effects of attentional control and emotion

Attentional control was not found to modulate activity in any region for neutral misses relative to hits (“neutral AB”). However, critically and in line with our predictions, it was found to modulate activity for both fearful misses relative to hits (“fear blinks”) and fear blinks relative to neutral blinks. Strong negative correlations with attentional control were observed in right DLPFC for fear blinks (cluster peak *x*, *y*, *z* = 45, 21, 21, *Z* = 3.09, Psvc = 0.04) and fear blinks relative to neutral blinks (cluster peak *x*, *y*, *z* = 42, 18, 21, *Z* = 3.6. Psvc = 0.01), respectively. These negative correlations were also observed in STS, for both fear blinks (cluster peak *x*, *y*, *z* = 45, −24, 0, *Z* = 3.16, Psvc = 0.04) and fear blinks relative to neutral blinks (cluster peak *x*, *y*, *z* = 45, −33, 0, Z = 3.01, Psvc = 0.05).

**Figure 4:**
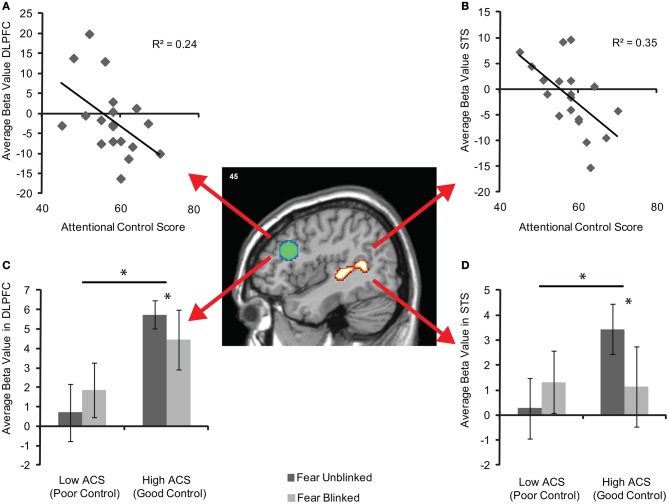
**Brain activity in the independently-defined DLPFC (green sphere based on previous coordinates) and STS (activity cluster based on localizer scan) regions of interest for fear blinked > unblinked trials. (A)** Shows average unstandardized beta values in DLPFC, and **(B)** in STS, across participants as a function of attentional control score across the entire sample (*n* = 19). **(C)** Shows mean (±S.E.) activity in DLPFC, and **(D)** in STS for each AB trial type of the high (*n* = 7) and low (*n* = 7) ACS groups. The significant main effects and interactions are marked with an asterisk.

To examine the strength of the relationships between activity in these areas and ACS whilst addressing the potential issues of non-independence (Kriegeskorte et al., 2010), average unstandardized beta values within each of the entire pre-defined ROIs were calculated for each individual. Significant correlations between ACS and DLPFC were observed for both fear blinks *r* = −0.40, df = 17, *p* = 0.04 and fear blinks relative to neutral blinks *r* = −0.49, df = 19, *p* = 0.02 (Figure 4A) and between ACS and STS, *r* = −0.57, df = 17, *p* = 0.006 for fear blinks and *r* = −0.591, df = 17, *r* = 0.004 for fear blinks relative to neutral blinks (Figure 4B).

Plots of high and low ACS groups suggest that the effects in both the DLPFC (Figure 4C) and STS (Figure 4D) are driven by relatively increased activity in the high ACS group when the target is perceived. Additionally whilst the high ACS group show reductions in activity when an item is blinked the low ACS group appear to show increases in activity. Following this up repeated-measures ANOVAs reveal significant performance by group interactions in both the DLPFC, *F*_(1, 11)_ = 4.42, pη^2^ = 0.29, *p* = 0.05, and STS *F*_(1, 11)_ = 10.28, pη^2^ = 0.48, *p* = 0.008, as well as a main effect of group in the DLPFC, *F*_(1, 11)_ = 5.31, pη^2^ = 0.33, *p* = 0.042. Paired samples *t*-tests revealed no significant change in activity between seen and unseen T2 target trials in either the DLPFC (*t* = −1.1, df = 5 *p* = 0.32) or STS (*t* = −1.95, df = 5, *p* = 0.11) in the low ACS group. However, the high ACS group showed significantly reduced activity in both DLPFC (*t* = 2.67, df = 6, *p* = 0.037) and STS (*t* = 2.63, df = 6, *p* = 0.039) on fear trials when the target was blinked.

#### Effects of anxiety

In line with our behavioral findings, trait anxiety was not found to modulate activity in any of our regions, for fear blinks, or fear blinks relative to neutral blinks. Furthermore, negative correlations between average beta values in DLPFC and STS and ACS for fear blinks relative to neutral blinks remained when controlling for both trait anxiety (HAD-A) (DLPFC *r* = −0.45, *p* = 0.03, df = 16, STS *r* = −0.57, *p* = 0.007, df = 16) and amygdala reactivity (DLPFC *r* = −0.47, *p* = 0.04, df = 15, STS *r* = −0.59, *p* = 0.007, df = 16).

#### Direct comparison of the influence of attentional control on DLPFC vs. amygdala activity

Finally a William's test comparing the size of the correlations between ACS and DLPFC reactivity with that between ACS and amygdala activity showed a trend to significance (*t* = 1.53, df = 15, *p* = 0.06) suggesting individual differences in ACS were more strongly correlated with DLPFC than amygdala activity.

### Whole brain analyses

Complementary whole brain analyses were carried out for each of the above contrasts. No significant activity was observed once whole brain correction was applied.

## Discussion

A wealth of research demonstrates attentional biases toward threat in anxiety disorders (Bar-Haim et al., 2007). The majority of research to date has focused on the role of trait negative emotionality (indexed by trait anxiety, behavioral inhibition, neuroticism, etc.) in such biases. There has been relative neglect of the role of individual differences in regulatory temperament dimensions, including attentional control (Posner and Rothbart, 2009), despite increasing evidence that variation in attentional control prospectively predicts the development and maintenance of mood and anxiety disorder symptomatology, both independently (additively), and in interaction with negative emotionality.

We found that variation in attentional control was related to attentional “disengagement” from threat. That is, people with lower ACS scores showed a selective enhancement of the AB following a threat-related vs. neutral T1. In concordance with this, activity in DLPFC was modulated by individual differences in attentional control for threat but not neutral ABs. Individuals with higher ACS scores showed greater DLPFC activity for unblinked threat trials, whilst the DLPFC did not show such a modulatory effect in those with lower ACS scores. We discuss these findings and their implications, in turn.

### Impact of variation in attentional control on temporal attention to threat

Theoretical accounts of the AB suggest that the reduction in T2 performance following T1 presentation is the result of transient increases in attentional demands required to allow for selection, working memory encoding, episodic registration and response selection of the T1 stimulus, meaning these resources are not available for redeployment to T2 at short T1–T2 intervals (Ward et al., 1997; Dux and Marois, 2009). de Jong et al. (2010) showed a larger AB following a threat related (vs. neutral) T1, that was independent of anxiety. They interpreted their findings as being consistent with an automatic prioritized processing of threat stimuli. However, whilst we too observe no effects of individual differences in anxiety on AB to threat, we found that a relatively larger AB for T1 threat stimuli was only seen in individuals with poor attentional control. In individuals with good attentional control, there was no advantage for threat-related T1 targets relative to neutral T1s in modulating the AB. Our findings are thus inconsistent with strong automaticity accounts of threat processing (see also Stein et al., 2010).

Findings by Stein et al. (2009) and Schupp et al. (2007) help clarify our results. Stein et al. found that the effect of fearful faces on the AB is task-dependent. When the emotional expression of the face stimuli had to be indicated, fearful faces induced a stronger AB than did neutral faces. However, with identical physical stimulation, the enhancement of the AB by fearful faces disappeared when participants had to judge face gender. They concluded that fearful faces attract more attentional resources, leaving less processing capacity for a T2 appearing at short T1–T2 intervals, but only when T1 emotion is selectively attended as part of current task goals. Consistent with this, Schupp et al. (2007) found, using high temporal resolution ERPs that implicit (task-irrelevant) emotion and explicit attention acted synergistically at later processing stages, but independently at perceptual encoding stages under RSVP conditions. Our results indicate that the emotional aspects of negative stimuli do not necessarily have to be explicitly task relevant in order to attract greater attentional resources in individuals with poor temperamental attentional control.

According to models of attentional control, such as biased competition (Desimone and Duncan, 1995) and its development in guided activation theory (Miller and Cohen, 2001), individual differences in interference from task irrelevant information arise from variation in the ability to actively maintain representations that guide control of tasks. These representations provide a top-down excitatory bias to groups of neurons processing task-relevant information. Because their activity is heightened relative to neurons processing task-irrelevant information, distracting information has less effect. Thus, we might expect that individuals with poorer attentional control may have difficulty in actively maintaining or implementing task representations (Posner et al., 2002). In the absence of strong top down control, we argue, these individuals are unable to prevent emotion potentiated attention effects during the capacity-limited later-stage processing underlying the AB (Woodman and Vogel, 2008; Martens et al., 2010). This difficulty appears relatively specific however: poor attentional control was not associated with reduced dual-task performance *per se*, only impacting on performance in the presence of emotional T1 targets. This is presumably because top-down excitatory biases are especially important for exerting attentional control when task-irrelevant information can effectively compete with task-relevant information [in this case face gender (Kaul et al., 2011)] for priority in processing. Such mechanisms may be particularly relevant in order to focus task demands on face gender, as opposed to expression, processing, since emotion expression processing is relatively automatic (Pessoa, 2005).

Here we did not observe any effects of anxious temperament on T2 performance following threat-related T1s (Georgiou et al., 2005; Koster et al., 2006), nor did attentional control interact with anxiety to predict performance (Derryberry and Reed, 2002; Lonigan and Vasey, 2009). Because many studies linking anxiety traits to attentional biases focused on individuals with high trait vulnerability (Bar-Haim et al., 2007) it may be that our modestly sized sample did not have sufficient range to ascertain relations between anxious temperament traits and attentional bias for threat. Also, our study may not have been adequately powered to detect interactions between anxiety and attentional control. Further, we did not use individual concern-specific (Mathews and MacLeod, 1985), but rather generic threat stimuli. Despite these limitations, it should be noted, however, that most previous studies did not routinely measure trait attentional control. One conclusion from our results is that variation in emotional interference from task-irrelevant threat does not result solely (or perhaps even primarily) from variation that is unique to trait negative emotionality. Theoretical models of individual differences in attentional bias for threat, therefore, need to take into account the overlap between attentional control and negative emotionality, which are related, but distinguishable, facets of self-control (Evans and Rothbart, 2009). Such a suggestion does not preclude the possibility that anxiety may uniquely influence other aspects of threat processing. According to Cisler and Koster (2010), attentional control ability underlies difficulty in “disengagement” from threat, whereas anxiety influences facilitation. Our findings are consistent with these proposals if disengagement is operationalized as sustained resource allocation to task-irrelevant threat.

Our findings are perhaps most consistent with the cognitive model of Mathews and Mackintosh (1998). On this account, a balance between opposing influences of an anxiety-linked threat-evaluation system and an independent task control system determines the extent of any attentional bias for task-irrelevant threat. In situations where threat evaluation is low, the major influence on attentional bias is likely to be variation in the strength of top-down task control. Our findings do not support, however, a recent account positing that trait anxiety, even in unselected populations, is directly linked to impoverished recruitment of attentional control mechanisms to inhibit distractor processing (Bishop, 2009).

### Role of prefrontal cortex in control of emotional interference

Neurally we observed a modulation of activity in the DLPFC for blinked relative to unblinked trials that was specific to the condition in which a threat T1 was present. Individuals with better attentional control showed reduced DLPFC activity for fear blinks, an effect not seen in those with lower ACS scores. These data are consistent with neural models of cognitive control (Miller and Cohen, 2001; Duncan, 2010). These models suggest that DLPFC maintains the representations that guide control of tasks, providing excitatory feedback to groups of neurons processing task relevant aspects of the stimulus and reducing the influence of distracting information. Thus, for those with better control, who were less susceptible to the emotional AB, a blink following a fearful face was associated with reduced activity in DLPFC, a region previously linked to variation in selective attention and task control (Polk et al., 2008; Leber, 2010).

Interestingly, if anything, the reverse appears to be the case in those with poorer control. In those with lower ACS scores, increases in prefrontal activity alongside corresponding increases in STS [a region which responds to threat expressions (Pessoa et al., 2002)] were observed for fear AB trials, compatible with the notion that despite DLPFC engagement these individuals may be less able to suppress the processing of the task irrelevant “threat” aspects of the face stimulus (compare Eysenck and Derakshan, 2011). In line with these findings, Schmitz et al. (2010) found that attentional selection was redistributed in older adults from posterior perceptual to goal-directed DLPFC mechanisms due to an age-related “leakiness” of early perceptual features and thus enhanced demands on late-stage selection processes.

A few previous studies have observed increased recruitment of DLPFC in the presence of irrelevant emotional distraction. Compton et al. (2003) found increased DLPFC activity during an emotional Stroop task, and argued that this was related to increased engagement of task control mechanisms in the face of emotional distraction (see also Denkova et al., 2010). However, that study did not look at individual differences in attentional control, or link activity to performance. Bishop et al. (2007) found that variation in attentional control (controlling for anxiety) was related to DLPFC activity to threat-related distractors under low (but not high) perceptual load, and linked DLPFC activity to late selection mechanisms, but no effects of attentional control on performance were seen in that study. Fales et al. (2008) found that depressed individuals showed reduced DLPFC activity when ignoring fear faces (relative to neutral) in a spatial attention paradigm, and a similar finding for high anxious individuals was reported by Bishop et al. (2004) (see also Most et al., 2006), but again, in the absence of anxiety effects on performance. Our findings are the first to demonstrate a clear role for DLPFC in attentional control over emotional interference effects, and link them to variation in temperamental attentional control.

Although not the primary focus of our experiment, and with the caveat that our fMRI sequence may not have been optimal to maximize signal from the amygdala, amygdala activity was not related to the presence of a threat-related AB. Further, we saw no modulation of amygdala activity for threat-related ABs by individual differences in attentional control or trait anxiety. The amygdala has often been considered to be a source of “emotional attention” underpinning attentional biases for threatening stimuli (Vuilleumier, 2005) and to underpin the influence of anxiety on such processing biases (Bishop et al., 2004), although previous studies have frequently found anxiety influences on the amygdala in the absence of performance effects.

It may be that the current paradigm (threat related T1) taps those aspects of attentional bias (“disengagement”) that are most strongly associated with prefrontal control mechanisms (Cisler and Koster, 2010). Paradigms that emphasize e.g., rapid shifts of attention to threat-stimuli may be more effective in engaging the amygdala, which might mediate facilitated attention to threat (Cisler and Koster, 2010; but see Tsuchiya et al., 2009). For example, Carlson et al. (2009) found amygdala activity related to spatial orienting to masked fearful faces in a dot-probe task. Using a related paradigm to the current one, in which threatening (or arousing) T2 stimuli follow a neutral T1, it has been found that AB effects are smaller for emotional vs. neutral T2 stimuli (Anderson, 2005), especially in anxious individuals (Fox et al., 2005), and this effect may result from more rapid early detection of threat. An initial study (Anderson and Phelps, 2001) found that lesions encompassing (but not restricted to) the amygdala abolish the advantage for emotional T2s (Anderson and Phelps, 2001; see also Schwabe et al., 2011 for complementary fMRI findings, who also found evidence that regions of prefrontal cortex including dorsal anterior cingulate mediated the influence of a threat T1). However, a recent study in more selective amygdala lesioned individuals failed to replicate this effect (Bach et al., 2011). Moreover, a few recent fMRI studies have, in addition to the amygdala, implicated regions of prefrontal cortex in potentiating performance for threat related T2 stimuli. Lim et al. (2009) found that the influence of the amygdala on visual cortical responses for fear conditioned T2 stimuli was mediated via regions of the medial frontal gyrus (see also De Martino et al., 2009). Most notably, Piech et al. (2011) recently found that amygdala lesions did not influence performance on an emotional AB task in which emotional distractors impair the detection of subsequent targets (Most et al., 2005), a task which is sensitive to individual differences in attentional control (Peers and Lawrence, 2009). Hence it appears that attentional regions in prefrontal cortex are closely linked to both the interfering effects of a threat T1 on a neutral T2, and potentially, though we did not address the issue in the current study, the advantage of a threat T2 in the AB.

## Concluding remarks

To conclude, we find that variation in prefrontal control mechanisms is related to variation in the sustained processing of task-irrelevant threat in an attentional dwell-time paradigm. The protective role of frontally driven attentional control against irrelevant threat in a non-clinical population fits well with findings from longitudinal studies, which suggest that individual differences in attentional control predict later psychological adjustment (e.g., Van Oort et al., 2011). Our findings suggest a possible mechanism by which attentional control could contribute to the development of resilience, and more generally speak to the importance of studying individual variation in neural mechanisms of attentional control.

### Conflict of interest statement

The authors declare that the research was conducted in the absence of any commercial or financial relationships that could be construed as a potential conflict of interest.

## Footnotes

^1^The robustness of these ROI's was examined by examining comparisons of the two single task conditions in the main experiment, these revealed the expected finding of significant activity in the FFA for the attend faces vs. attend scenes, and significant activity in the PPA and early VC for the reverse contrast.
